# Histological, immunohistochemical and serological investigations of the ovary during follicular phase of estrous cycle in Saidi sheep

**DOI:** 10.1186/s12917-024-03933-z

**Published:** 2024-03-09

**Authors:** Mahmoud Abd-Elkareem, M. A. Khormi, Ragab Hassan Mohamed, Fatma Ali, Mervat S. Hassan

**Affiliations:** 1 Department of Cell and Tissues, Faculty of Veterinary Medicine, Assiut University, Assiut, 71526 Egypt; 2 Department of Biology, College of Science, Jazan University, P.O. Box. 114, Jazan, 45142 Kingdom of Saudi Arabia; 3 Theriogenology Department, Faculty of Veterinary Medicine, Aswan University, Aswan, Egypt; 4 Physiology Department, Faculty of Veterinary Medicine, Aswan University, Aswan, Egypt; 5 Theriogenology Department, Faculty of Veterinary Medicine, New-Valley University, New Valley, 725211 Egypt

**Keywords:** Saidi sheep, Ovary, Ovarian follicles, Estrous cycle, SOD2, Antioxidants, PRA

## Abstract

**Background:**

Saidi sheep are the most abundant ruminant livestock species in Upper Egypt, especially in the Assiut governorate. Sheep are one of the most abundant animals raised for food in Egypt. They can convert low-quality roughages into meat and milk in addition to producing fiber and hides therefore; great opportunity exists to enhance their reproduction. Saidi breed is poorly known in terms of reproduction. So this work was done to give more information on some hormonal, oxidative, and blood metabolites parameters in addition to histological, histochemical and immunohistochemical investigations of the ovary during follicular phase of estrous cycle. The present study was conducted on 25 healthy Saidi ewes for serum analysis and 10 healthy ewes for histological assessment aged 2 to 5 years and weighted (38.5 ± 2.03 kg).

**Results:**

The follicular phase of estrous cycle in Saidi sheep was characterized by the presence of ovarian follicles in different stages of development and atresia in addition to regressed corpus luteum. Interestingly, apoptosis and tissue oxidative markers play a crucial role in follicular and corpus luteum regression. The most prominent features of the follicular phase were the presence of mature antral (Graafian) and preovulatory follicles as well as increased level of some blood metabolites and oxidative markers. Here we give a new schematic sequence of ovarian follicles in Saidi sheep and describing the features of different types. We also clarified that these histological pictures of the ovary was influenced by hormonal, oxidative and blood metabolites factors that characterizes the follicular phase of estrous cycle in Saidi sheep.

**Conclusion:**

This work helps to understanding the reproduction in Saidi sheep which assist in improving the reproductive outcome of this breed of sheep. These findings are increasingly important for implementation of a genetic improvement program and utilizing the advanced reproductive techniques as estrous synchronization, artificial insemination and embryo transfer.

## Background

Sheep are widely bred livestock in the world as it providing a large amount of meat, fat, milk, wool and other products for humans. Increasing animal reproductive performance and the litter size in sheep has always been the focus of many breeding research [1]. The Saidi breed of the Egyptian sheep has a long fat tail and a generally dark brown fleece. Its breeding area is in the Upper Egypt, South of Assiut and it was considered as the oldest of the Egyptian breeds [2]. Due to its high conception (82- 92%) and twining (1.5%) rates the demand for this breed increases [3, 4] Saidi ewes were a breed of sheep with nearly no reproductive seasonality where the estrous cycles distributed along the year although there was a decline in the estrous and ovarian activity during spring [5].

Ovary in mammals is responsible for the development, maturation, and release of mature oocytes for fertilization, as well as for the synthesis and secretion of many hormones and factors that are important for follicular development, menstrual/estrous cyclicity, and maintenance of the integrity and function of the reproductive tract [6–8]. The ovary is female’s animal reproductive organ that contains a large number of follicles at different developmental stages from primordial to mature follicles [9–11]. The development of follicles shows a dynamic process of ‘follicular waves’ including recruitment, selection, dominance, turnover (atresia) or ovulation [12].

In sheep and cattle, recruitment is the growth of many antral (small and large) follicles and is the gonadotrophin dependent stage, selection is the adjustment of the number of recruited follicles to the number of ovulations followed by dominance and maturation of the selected ones. This cycle of recruitment, selection and dominance occur at regular intervals whatever the reproductive stage (prepuberal, follicular, luteal phase, anoestrus) and this controlled by endocrine, paracrine and autocrine signals [12–16]. Follicular development is regulated by a variety of hormones like FSH, LH inhibin, progesterone and estrogen, and the imbalance of these hormone expression levels can disrupt the follicular development affecting animal reproduction and fertility [1, 17–19].

Oxidative stress arises from an imbalance between pro-oxidants (free radical species) and the body's scavenging ability (antioxidants). Oxidative stress has been considered a contributing factor to many female reproductive disorders including infertility, endometriosis and polycystic ovarian syndrome [20]. Levels of oxidative stress in the follicular fluid may have a fundamental impact on oocyte survival and thereby its outcomes [21]. It was found that reactive oxygen species modulates multiple physiological processes including: oocyte maturation, fertilization, embryo development, pregnancy, normal parturition and in initiation of preterm labor [22].

There was a lack of sufficient information about the follicular development in saidi sheep and its relation to hormonal and oxidative influences. So, the aim of the present study was to give more details on the follicular dynamics and its relation to some serological parameters in Saidi sheep during the follicular phase of the estrous cycle which may help in their implications for reproductive management.

## Materials and methods

### Ethical statement

The experimental protocol was approved by the Local Ethical Committee and by the Institutional Review Board of Molecular Biology Research and studies Institute, Assiut University (22–2023-0028) and was carried out in accordance with relevant guidelines and regulations. This research was done in compliance with the ARRIVE guidelines and regulations (https://arriveguidelines.org). All national and institutional guidelines for animal care and use have been followed throughout the study procedures.

### Animals and samples

Biological samples were collected from apparently healthy slaughtered (according to Islamic religion) Saidi sheep aged 2 to 5 years and weighted (38.5 ± 2.03 kg) at Assiut abattoir, Assiut governorate, Egypt.

### Blood samples collection

Blood samples were obtained from jugular vein of ewe (*n* = 25) before slaughtering during follicular phase of estrous cycle in blood collection tubes [23]; then centrifuged at 3000 rpm for 10–15 min. The separated serum was stored in a deep freezer at -20°C until use.

### Serum biochemical assay

Based on the manufacturer's instructions, calometeric kits of Blood glucose (BG), cholesterol (TC), urea and total protein (TP) were purchased from Diamond (Egypt). Kinetic kits of aspartate aminotransferase (AST) and alanine aminotransferase (ALT) were supplied from the Human Company (Germany) [24].

### Steroid hormone and Oxidative stress parameters

Serum Progesterone (P4) & Estrogen (E2) levels were obtained using an enzyme linked immunosorbent assay (ELISA) kit provided by Bio Check, Inc., South San Francisco, USA (Catalog number; BC-1113). Kits for malonaldhyde (MDA), nitric oxide (NO) and total antioxidant capacity (TAC) were obtained from Biodiagnostics Company (Egypt) [25].

### Histological examination

Ovaries (n = 10) were fixed in 10% neutral buffered formalin. The formalin-fixed ovaries were dehydrated in ascending grades of ethanol, cleared in methyl benzoate, and embedded in paraplast. Paraffin sections at 5 μm in thickness were cut and stained with the following histological stains:

1. Haematoxylin and Eosin for general histological examination of the ovary [26].
2. Periodic acid Schiff (PAS) technique for demonstration of glycoprotein [26].
3. Crossmon’s trichrome technique for staining collagen fibers [27].
4. Picro-Sirius red technique to differentiate between mature and immature collagen fibers [28, 29].
5. Orcien stain for detection of distribution of elastic fibers in the ovary [30].

All staining slides were examined by an Olympus BX51 microscope and the photographs were taken by an Olympus DP72 camera adapted into the microscope.

### Oxidative stress detection

#### Immunohistochemistry of glutathione reductase and superoxide dismutase 2

Paraffin-embedded tissue sections were deparaffinized, rehydrated, and rinsed in phosphate buffered saline (PBS). Then slides were placed in 10 mM sodium citrate buffer (pH 6.0) for antigen retrieval at (95–98 °C) in a water bath for 20 min. Endogenous peroxidase was blocked by incubating the slides in 3% hydrogen peroxide for 10 min at room temperature. This followed by washing the slides in PBS (3 times for 5 min each). The sections were incubated overnight in a humid chamber with rabbit polyclonal antibodies. For immunohistochemical detection of glutathione reductase (GR) and superoxide dismutase 2 (SOD2) in the ovary, we used polyclonal anti-glutathione reductase and anti-superoxide dismutase 2 antibodies, respectively (Chongqing Biospes Co., Ltd, China) and Power-Stain™ 1.0 Poly horseradish peroxidase (HRP) DAB Kit (Genemed Biotechnologies, Inc, 458 Carlton Ct. South San Francisco, CA 94080, USA) [31].

### Hormonal receptor detection

#### Immunoexpression of progesterone receptor alpha

The protocol used was according to the company instructions and as our previous study [17]. The fixed ovaries were dehydrated in ethanol, cleared in methyl benzoate and then embedded in paraplast. Sections (5 μm) of paraplast-embedded tissue were dewaxed by xylene. Subsequently, rehydration by 100%, 95%, 80% and 70% ethanol, and slides were rinsed in PBS (pH 7.4). Endogenous peroxidase were prevented by adding 3% hydrogen peroxide followed by washing in PBS. For antigen detections, the slides were placed in 10 mM sodium citrate buffer (pH 6.0) at (95–98 °C) in a water bath for 20 min followed by cooling at room temperature. Sections were then rinsed in PBS. Immunoexpression of progesterone receptor alpha was done by using; progesterone receptor rabbit pAb (Catalog No.: A0321), ABclonal, USA. Sections were then incubated with the primary antibodies for 30–60 min at room temperature. The slides were washed with PBS then follow the company instructions of poly Q stain 2 step detection system goat anti-mouse/rabbit HRP, peroxidase quench, DAB kit, quartett, Germany. The sections were counterstained in Harris hematoxylin for 30 s. Then sections were dehydrated by ethanol 95%, and ethanol 100%, cleared of in xylene, and mounted with DPX.

### Apoptosis detection

#### TUNEL assay

Detection of apoptosis was done using In Situ Cell Death Detection Kit, Fluorescein (Sigma-Aldrich, USA). Terminal deoxynucleotidyl transferase (TdT) dUTP Nick-End Labeling (TUNEL) assay was designed to detect apoptotic cells that undergo extensive DNA fragmentation during the late stages of apoptosis. This method was depending up on the ability of TdT to label blunt ends of double-stranded DNA breaks independent of a template. The protocol we used as the previous published protocol [32]. Slides were rinsed with PBS and directly analyzed under a fluorescence microscope.

## Results

### Physiological parameters during the follicular phase of the estrous cycle in saidi sheep

During follicular phase of ewe reproductive cycle; the serum hormonal profile (Mean ± SE) of P4 and E2 were (0.86 ± 0.06 ng/ml and 2.32 ± 0.08 pg/ml; respectively). The serum metabolic adaptation for (BG, TC, TP and serum urea) were (57.59 ± 13.94 mg/dl, 85.34 ± 28.69 mg/dl, 16.91 ± 0.90 g/dl and 5.67 ± 0.51 mg/dl; respectively). The changes of anti-oxidative/ pro-oxidative parameters during this phase were 7.30 ± 0.44 (nmol/ml) for MDA; 0.46 ± 0.02 umol/L for NO and 1.44 ± 0.19 Mm/ml for TAC. Data indicated that the serum liver enzymes were 128.41 ± 6.75 lU/L for AST activities and 67.68 ± 6.39 lU/L for ALT activities (Table 1).

**Table 1:** Serum biochemical reference ranges for ewes, including energy, protein and enzyme related metabolites during follicular phase of estrous cycle

Physiological –parameters	Unit	Study Data
**Steroid hormones**		
Progesterone	ng/ml	0.86 ± 0.06
Estrogen	pg/ml	2.32 ± 0.08
**Energy-related metabolites**		
Blood glucose	mg/dl	57.59 ± 13.94
Cholesterol	mg/dl	85.34 ± 28.69
**Protein-related metabolites**		
Total protein	g/dl	16.91 ± 0.90
Serum urea	mg/dl	5.67 ± 0.51
**Enzyme-related metabolites**		
AST	U/L	128.41 ± 6.75
ALT	U/L	67.68 ± 6.39
**Oxidative and antioxidtive parameters**		
MDA	nmol/ml	7.30 ± 0.44
NO	µmol/L	0.46 ± 0.02
TAC	Mm/ml	1.44 ± 0.19

*AST* Aspartate aminotransferase, *ALT* Alanine aminotransferase, Malonaldhyde (*MDA*), Total antioxidant capacity (*TAC*), Nitric oxide (*NO*)

### Histological features of the sheep ovary during the follicular phase of the estrous cycle

Our results revealed that the ewe ovary during the follicular phase of the estrous cycle contained many follicles in different stages of development and atresia in addition to regressed corpus luteum (corpus albicans). The ovary in ewe was consisted of an outer cortex and an inner medulla. The ovarian cortex was covered by ovarian surface epithelium which formed of single layers of flattened epithelial cells. Tunica albugenia of dense irregular connective tissue separated the ovarian surface epithelium from the developing follicles. Primordial follicles were located in the most peripheral zone of the cortex under the tunica albugenia. They were present in cell nests formed of more than one follicle. Each primordial follicle was formed of oogonium surrounded by single layer of flattened follicular cells. As the primordial follicles activated it transformed into primary follicles. The activated follicle was formed of oocyte surrounded by intermingled flattened and cuboidal follicular cells. The primary follicle was formed of oocyte surrounded by single layer of cuboidal follicular cells.

The growing follicles were formed of primary occyte surrounded by more than one layer of follicular cells. The progressive development and growth of the primary follicle included: changes in the oocyte and change in the follicular cells. The changes in the oocyte during follicular growth included: the occyte increased in size, Yolk granules appeared in the cytoplasm of the oocyte and Zona pellucida appeared around the oocyte; zona pellucida was a glycoprotein secreted by the surrounding follicular cells and occyte itself. Zona pellucida was a homogenous acidophilic PAS and light green positive membrane. Change in the follicular cells included: the flattened follicular cells change into cuboidal or columnar cells. The cuboidal cells were divided by mitotic divisions, increased in number and formed a stratified epithelium around the oocyte. The small antral follicles were growing follicles contained one or more small pools filled with follicular fluid.

**Fig. 1:**
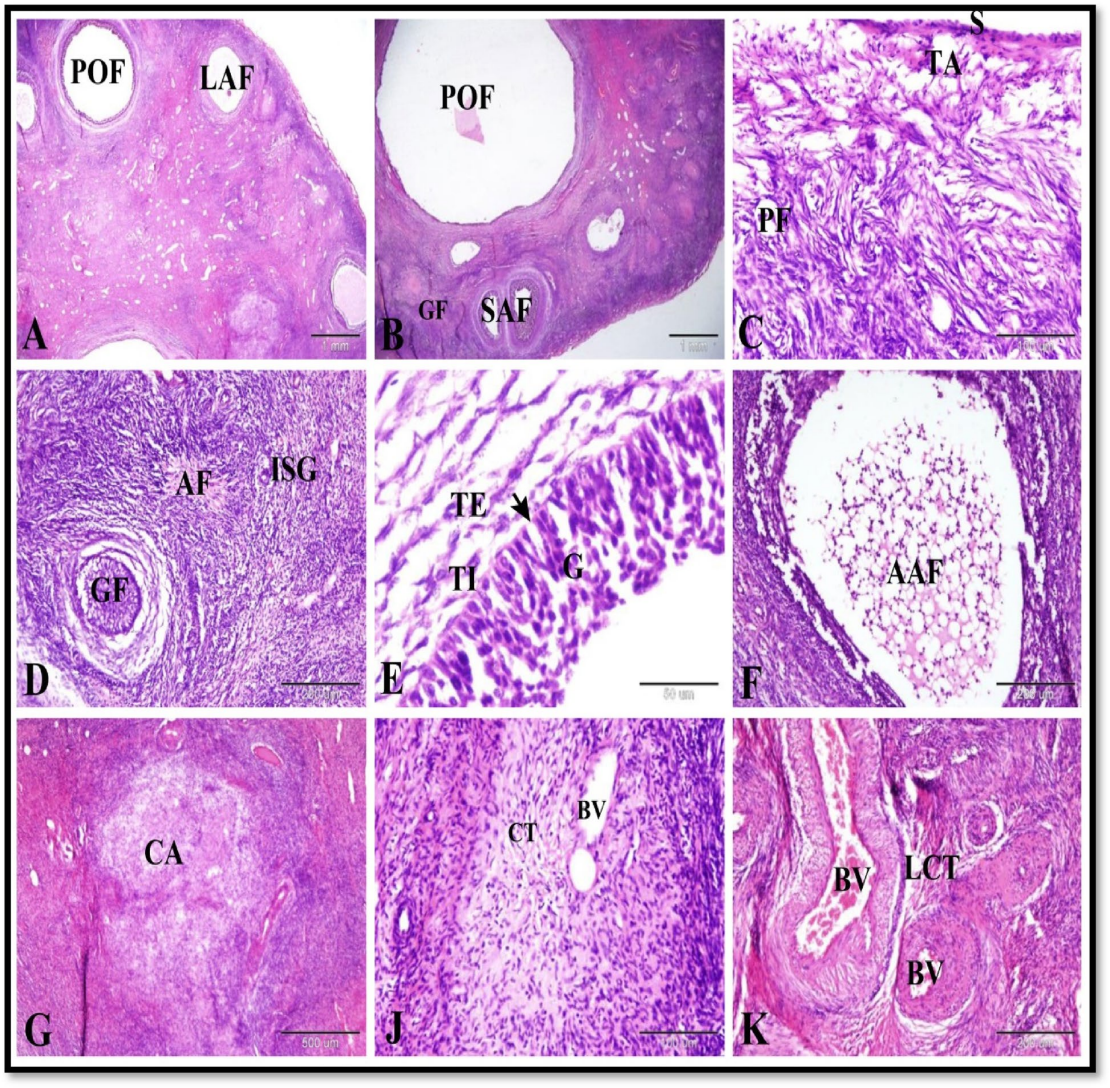
Photomicrograph of the sheep ovary during the follicular phase. **A** Ovarian cortex showing preovulatory follicles (POF) and large antral follicles (LAF). **B** Ovarian cortex showing growing follicles (GF), small antral follicles (SAF) and preovulatory follicles (POF). **C** Ovarian cortex showing ovarian surface epithelium (S) which formed of single layers of flattened epithelial cells, dense irregular connective tissue of tunica albugenia (TA) and primordial follicles (PF). **D** Ovarian cortex showing growing follicles (GF), atretic follicles (AF) and interstitial glands (ISG). **E** The wall of preovulatory follicles showing dissociated granulosa cells (G), thin basement membrane (arrow), theca interna (TI) and theca externa (TE). **F** Showing atretic antral follicle (AAF). **G** Showing corpus albicans (CA). **J** Showing the structure of the corpus albicans which composed of dense irregular connective tissue (CT) and some blood vessels (BV). **K** Showing the ovarian medulla formed of loose connective tissue (LCT) and numerous blood vessels (BV). Original magnification; **A** & **B**: X12.5, scale bar = 1mm, **C** & **J**: X200, scale bar = 100 µm, **D**, **F** & **K**: X100, scale bar = 200 µm, **E**: X 400, scale bar = 50 µm, **G**: X 40, scale bar = 500 µm, haematoxylin and eosin stain

The mature ovarian follicles (Graafian follicles) were the large antral follicles and they were formed of the following layers from inside to outside: large secondary occyte, zona pellucida (homogenous envelop, corona radiata (columnar cells), zona granulosa (stratified follicular epithelium surrounding single large follicular cavity filled with follicular fluid), cumulus oophorus (groups of cells which connected the ovum and its surroundings with the zona granuolsa), follicular basement membrane, theca interna (large spindle-shaped cells with epithelioid appearance), theca externa (less cellular, less vascular, more fibrous). The preovulatory follicles were large antral follicle with a very large single antrum and thin wall and bulging from the ovarian surface. The wall of preovulatory follicles showed dissociated granulosa cells, thin follicular basement membrane, theca interna and theca externa. In addition the ovaian cortex in ewe during follicular phase contained atretic antral follicles, corpus albicans and interstitial glands. The corpus albicans was composed of dense irregular connective tissue and some blood vessels. The ovarian medulla formed of loose connective tissue and numerous blood vessels (Fig. 1).

**Fig. 2:**
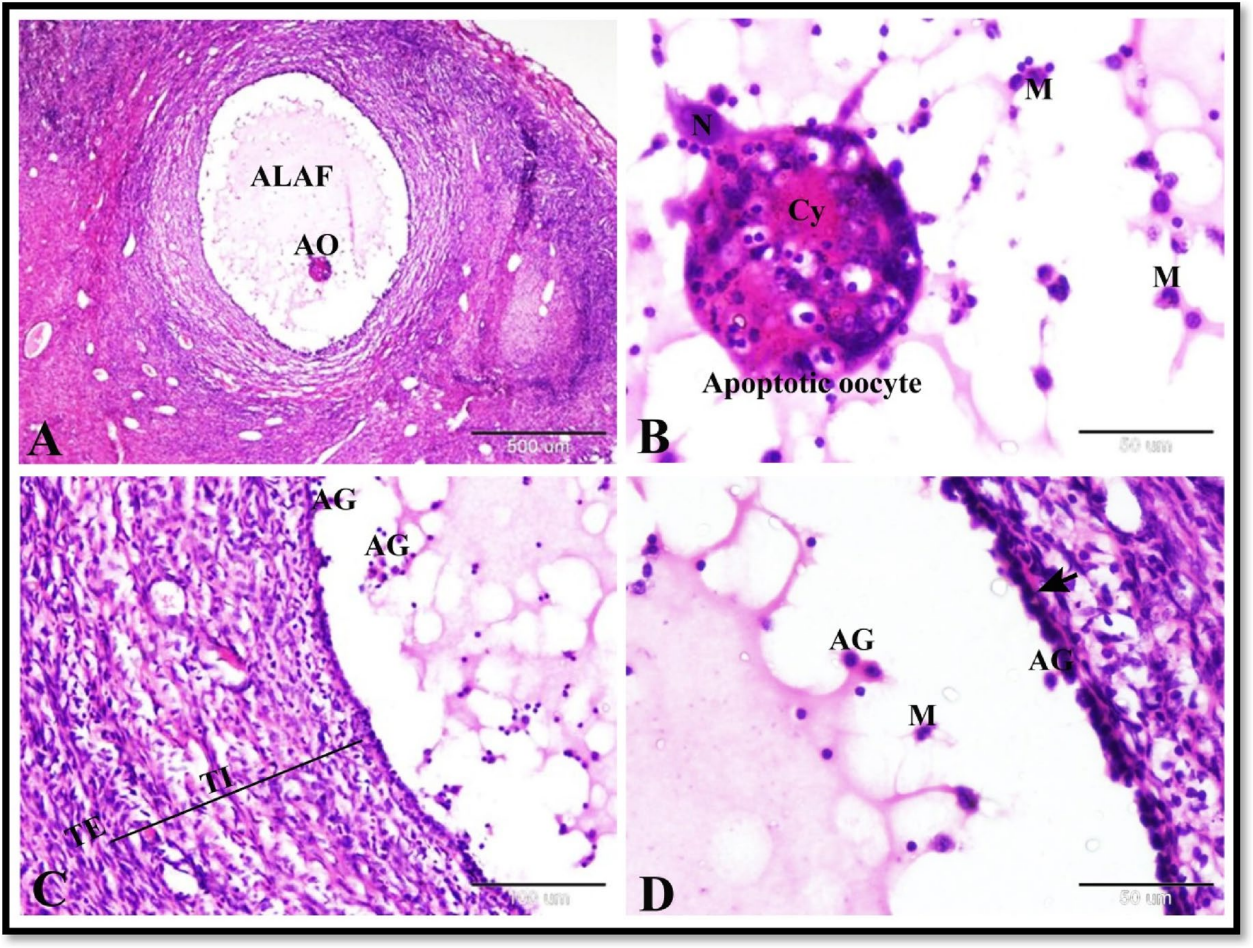
Photomicrograph of the sheep ovary during the follicular phase showing follicular atresia. **A** Showing atretic large antral follicle (ALAF) with apoptotic oocyte (AO). **B** Higher magnification of apoptotic oocyte in **A**; the oocyte have deeply stained acidophilic cytoplasm (Cy) and fragmented pyknotic nucleus (N) and surrounded by several macrophages (M). **C** & **D** Showing atretic large antral follicle with thickening and proliferation of theca interna (TI), apoptotic granulosa cells (AG), recruitment of several macrophages (M), thin interrupted follicular basement membrane (arrow) and normal theca externa (TE). Note the zona granulosa formed of single layer. Original magnification; **A**: X40, scale bar = 500 µm and **B**-**D**: X400, scale bar = 50 µm, haematoxylin and eosin stain

Herein we found that the follicular atresia in the sheep ovary during the follicular phase of estrous cycle were most prominent in the antral follicles especially the large antral ones. Atretic large antral follicle was characterized by the presence of apoptotic oocyte; the oocyte have deeply stained acidophilic cytoplasm and fragmented pyknotic nucleus and surrounded by several macrophages (Fig. 2A and B). It also characterized by thickening and proliferation of theca interna, apoptotic granulosa cells, recruitment of several macrophages, thin interrupted follicular basement membrane and normal theca externa. In some atretic large antral follicle, zona granulosa became formed of single layer (Fig. 2C and `D).

### Histochemical features of the sheep ovary during the follicular phase of the estrous cycle

**Fig. 3:**
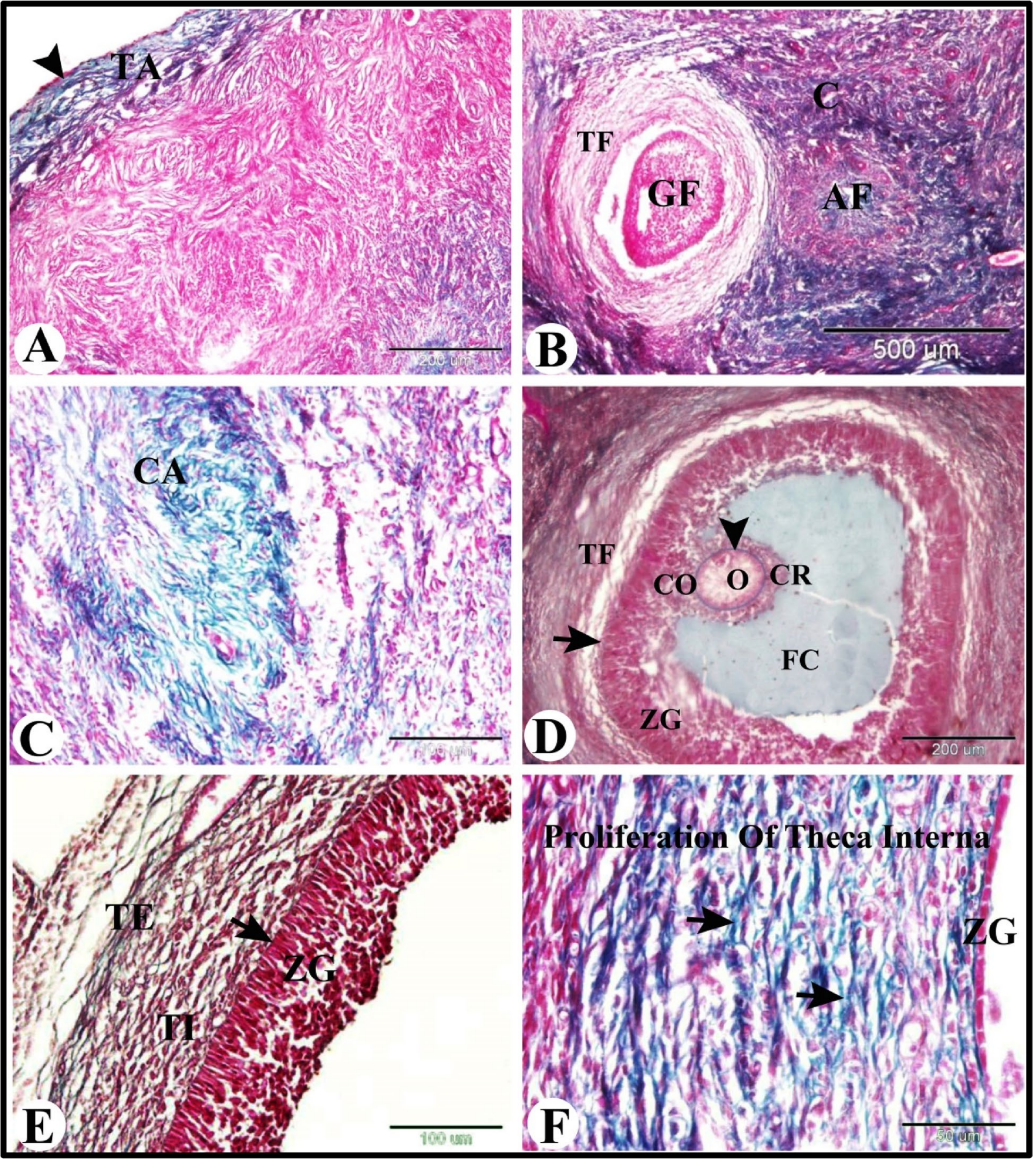
Photomicrograph illustrating the collagen fibers distribution in the sheep ovary during the follicular phase. **A** Showing ovarian surface epithelium (arrowhead) and tunica albuginea formed of dense irregular collagenous connective tissue. **B** Showing atretic follicle with a collagenous connective tissue core (AF), growing follicle (GF) surrounded by theca folliculi with few collegen fibers, and loose connective tissue of ovaian cortex with collgen fibers (C). **C** Showing corpus albicans (CA) which composed of dense irregular collegen fibers. **D** & **E** Showing mature graafian follicle which consisted of oocyte (O), zona pellucida (arrowhead), corona radiate (CR), cumulus oopherous (CO), large follicular cavity (FC) filled with follicular fluid, zona granulosa (ZG), Basement membrane (arrow), theca folliculi (TF) which formed of theca interna (TI) and theca externa (TE) with many collagen fibers. **F** Showing atretic anteral follicle with thinning of zona granulosa (become single layer) and thickening and proliferation of theca interna with proliferation of collagen fibers (arrow). Original magnification; **A** & **D**: X100, scale bar = 200 µm, **B**: X40, scale bar = 500 µm, **C** & **E**: X200, scale bar = 100 µm, **F**: X 400, scale bar = 50 µm, Crossmon's trichrome technique

We used Crossmon's trichrome technique to illustrate the collagen fibers distribution in the sheep ovary during the follicular phase of estrous cycle. We observed that the tunica albuginea formed of dense irregular collagenous connective tissue (Fig. 3A). Growing follicle were surrounded by theca folliculi with few collegen fibers and some atretic follicles were had collagenous connective tissue core. The loose connective tissue of ovaian cortex contained collgen fibers (Fig. 3B). The corpus albicans was composed of dense irregular collegen fibers (Fig. 3C). Theca externa of the mature graafian follicle also contained many collagen fibers. With Crossmon's trichrome technique zona pellucida and the follicular basement membrane were appeared green (Fig. 3D and E). The theca interna of atretic anteral follicle were characterized by proliferation of collagen fibers (Fig. 3F).

**Fig. 4:**
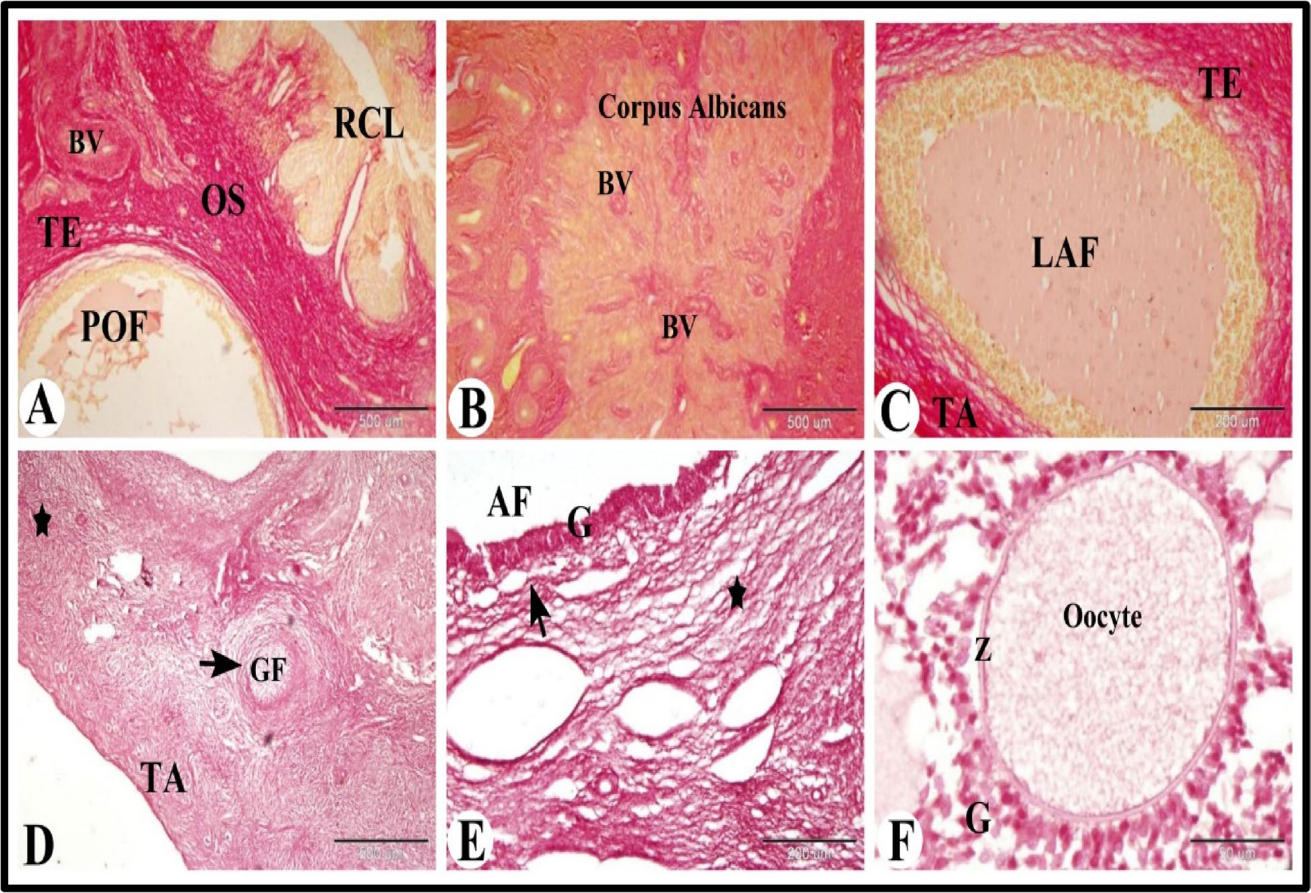
Photomicrograph illustrating the collagen fibers type I (A-C) and elastic fibers (D-F) distribution in the sheep ovary during the follicular phase. **A**-**C**: Showing collagen fibers type I in tunica albuginea (TA), theca externa (TE) of large antral follicle (LAF), and of preovulatory follicle (POF), surrounded the regressed corpus luteum (RCL), in corpus albicans (CA), surrounded the blood vessles (BV) and in ovarian stroma (OS). **D**-**F**: Showing elastic fibers in tunica albuginea (TA), ovarian stroma (  ) theca externa (arrow) of growing (GF) and antral follicles (AF). Note the orcein positive granulosa cells (G) of antral follicles and the orcein negative zona pellucida (Z) and the cytoplasm of the oocyte. Original magnification; **A**, **B** & **D**: X40, scale bar = 500 µm, C & E: X100, scale bar = 200 µm, F: X 400, scale bar = 50 µm, **A**-**C**: Picro-Sirius red technique and **D**-**F**: Orcien stain

To examine the distribution of the type I collagen fibers in the sheep ovary during the follicular phase of estrous cycle we used Sirius red stain. We found that the type I collagen fibers was located in tunica albuginea, in theca externa of large antral follicle and of preovulatory follicle, surrounded the regressed corpus luteum, in corpus albicans, surrounded the blood vessles and in ovarian stroma (Fig. 4A-C).

Whereas to examine the presence of elastic fibers in the sheep ovary during the follicular phase of estrous cycle we used Orcien stain. We found that few elastic fibers (dark red or brown) were present in tunica albuginea, ovarian stroma, theca externa of growing and antral follicles. We also observed that the granulosa cells of the antral follicles were orcein positive and the cytoplasm of the oocyte and zona pellucida were orcein negative (Fig. 4D-F).

**Fig. 5:**
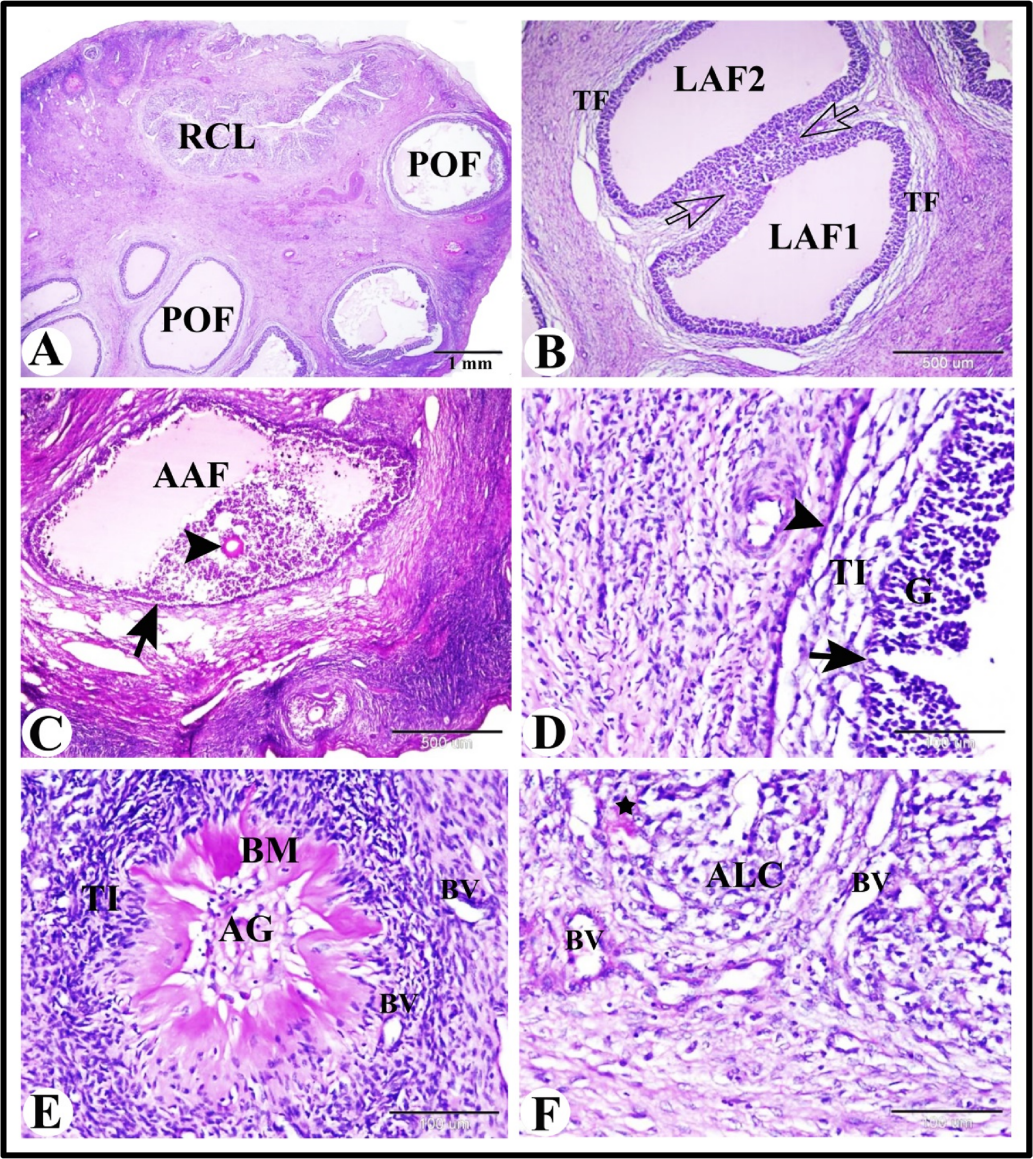
Photomicrograph of the sheep ovary during the follicular phase. **A** Ovarian cortex showing preovulatory follicles (POF) and regressed corpus luteum (RCL). **B** Ovarian cortex showing two large antral follicles (LAF1 & LAF2) surrounded by the same theca folliculi (TF) and connected by zona garnulosa with the same basement membrane (arrows). **C** Ovarian cortex showing atretic antral follicles (AAF) with thickening in the PAS positive follicular basement membrane (arrow) and zona pellucida (arrowhead). **D** The wall of preovulatory follicles showing dissociated granulosa cells (G), thin interrupted PAS positive follicular basement membrane (arrow), theca interna (TI) and theca externa (TE) with proliferation of fibroblasts (arrowhead). **E** Showing atretic antral follicle with hyalinization of the follicular basement membrane (BM), proliferation of theca interna cells (TI) and angiogenesis (BV). Note the central region contained few apoptotic granulosa cells (AG) and eliminated oocyte. **F** Showing regressed corpus luteum with autophagic lutein cells (ALC); some had PAS positive material (star) and degeneration of blood vessels with thick PAS positive endothelial basement membrane (BV). Original magnification; **A**: X12.5, scale bar = 1mm, **B** & **C**: X40, scale bar = 500 µm, **D**-**F**: X200, scale bar = 100 µm, PAS & Hx

To evaluate the distribution of the glycoprotein in the sheep ovary during the follicular phase of estrous cycle we used PAS technique. We demonstrated that the glycoprotein present in the PAS positive follicular basement membrane and zona pellucida of the growing and antral follicles (Fig. 5A-C). The preovulatory follicles showed thin interrupted PAS positive follicular basement membrane with proliferation of fibroblasts (Fig. 5D). In early stage of the antral follicles atresia the PAS positive follicular basement membrane and zona pellucida became thickened. While in the late (terminal) stage of the antral follicles atresia the PAS positive follicular basement membrane became hyalinized and formed collapsed glassy membrane which surrounded central region contained few apoptotic granulosa cells and eliminated oocyte. In addition to proliferation of theca interna cells with new angiogenesis peripheral to collapsed glassy membrane (Fig. 5E). Whereas the regressed corpus luteum was characterizes by autophagic lutein cells and some of lutein cells had PAS positive material and degeneration of blood vessels with thick PAS positive endothelial basement membrane (Fig. 5F).

### GR immunoexpression in the sheep ovary during the follicular phase of estrous cycle

**Fig. 6:**
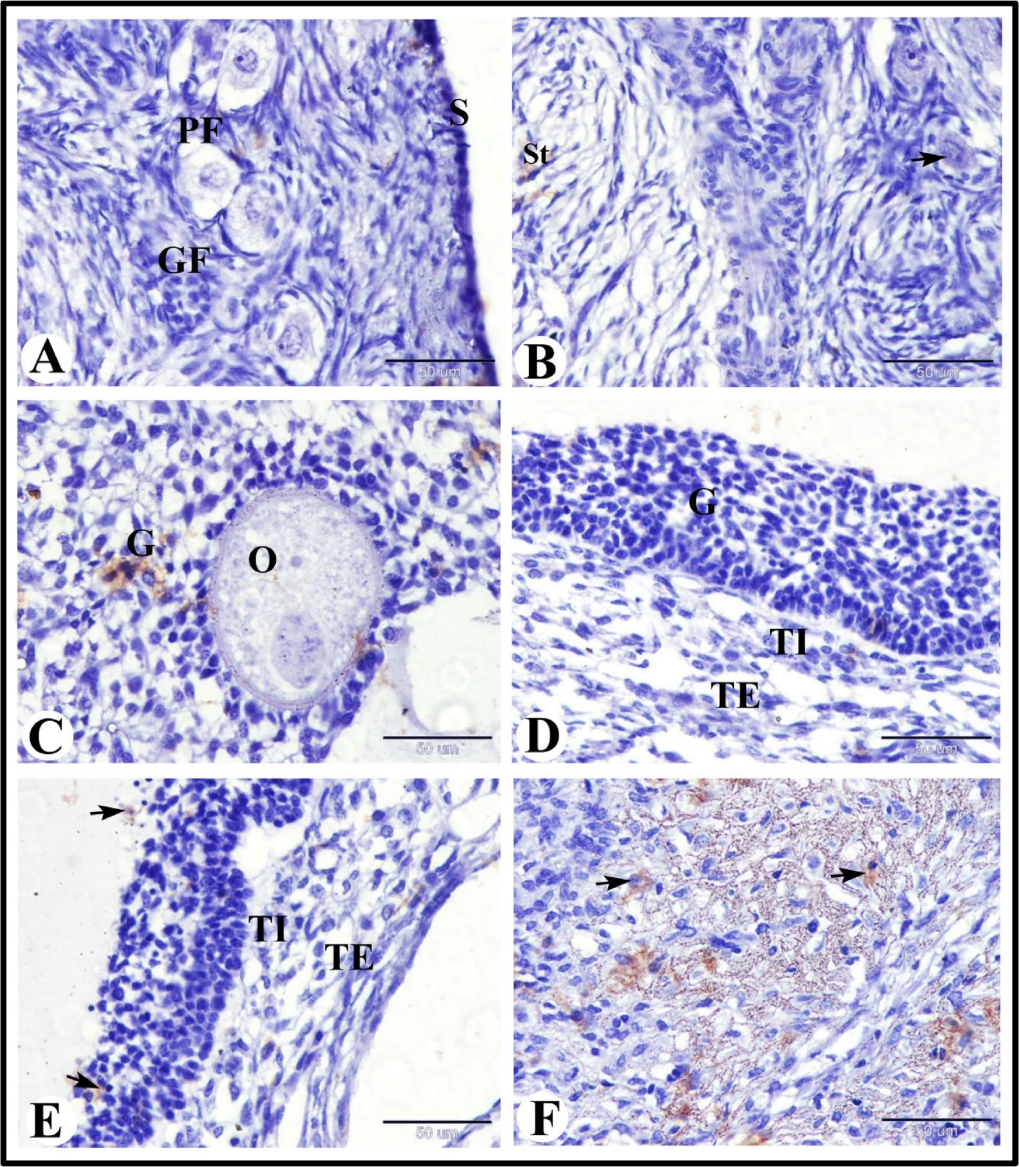
Photomicrograph of GR immunostaining in the sheep ovary during the follicular phase. **A** Showing slight GR immunostaining in the ovarian surface epithelium (S), negative GR immunostaining in the primordial follicles (PF) and growing follicle (GF). **B** Showing slight GR immunostaining in the stroma cells (St) and oocyte of the primary follicle (arrow). **C** Showing moderate GR immunostaining in granulosa cells (G) and negative GR immunostaining in oocyte (O) of the antral follicles. **D** Showing negative GR immunostaining in the granulosa cells (G), theca interna cells (TI) and theca externa cells of mature graafian follicles. **E** Showing moderate GR immunostaining in the apoptotic granulosa cells (arrows) and negative GR immunostaining in the theca interna cells (TI) and theca externa cells of atretic antral follicles. **F** Showing strong GR immunostaining in the lutein cells (arrows) of the regressed corpus luteum. Original magnification; **A**-**F**: X400, scale bar = 50 µm

Our study showed that there were slight GR immunoexpression in the ovarian surface epithelium, in the stroma cells and in the oocyte of the primary follicle and negative GR immunostaining in the primordial follicles and growing follicle (Fig. 6A and B). Moderate GR immunostaining in granulosa cells and negative GR immunostaining in oocyte of the antral follicles was observed (Fig. 6C). Negative GR immunostaining in the granulosa cells, theca interna cells and theca externa cells of mature graafian follicles (Fig. 6D). While there were moderate GR immunostaining in the apoptotic granulosa cells and negative GR immunostaining in the theca interna cells and theca externa cells of atretic antral follicles (Fig. 6E). Whereas there was strong GR immunoexpression in the lutein cells of the regressed corpus luteum (Fig. 6F).

### SOD2 immunolocalization in the sheep ovary during the follicular phase of estrous cycle

**Fig. 7:**
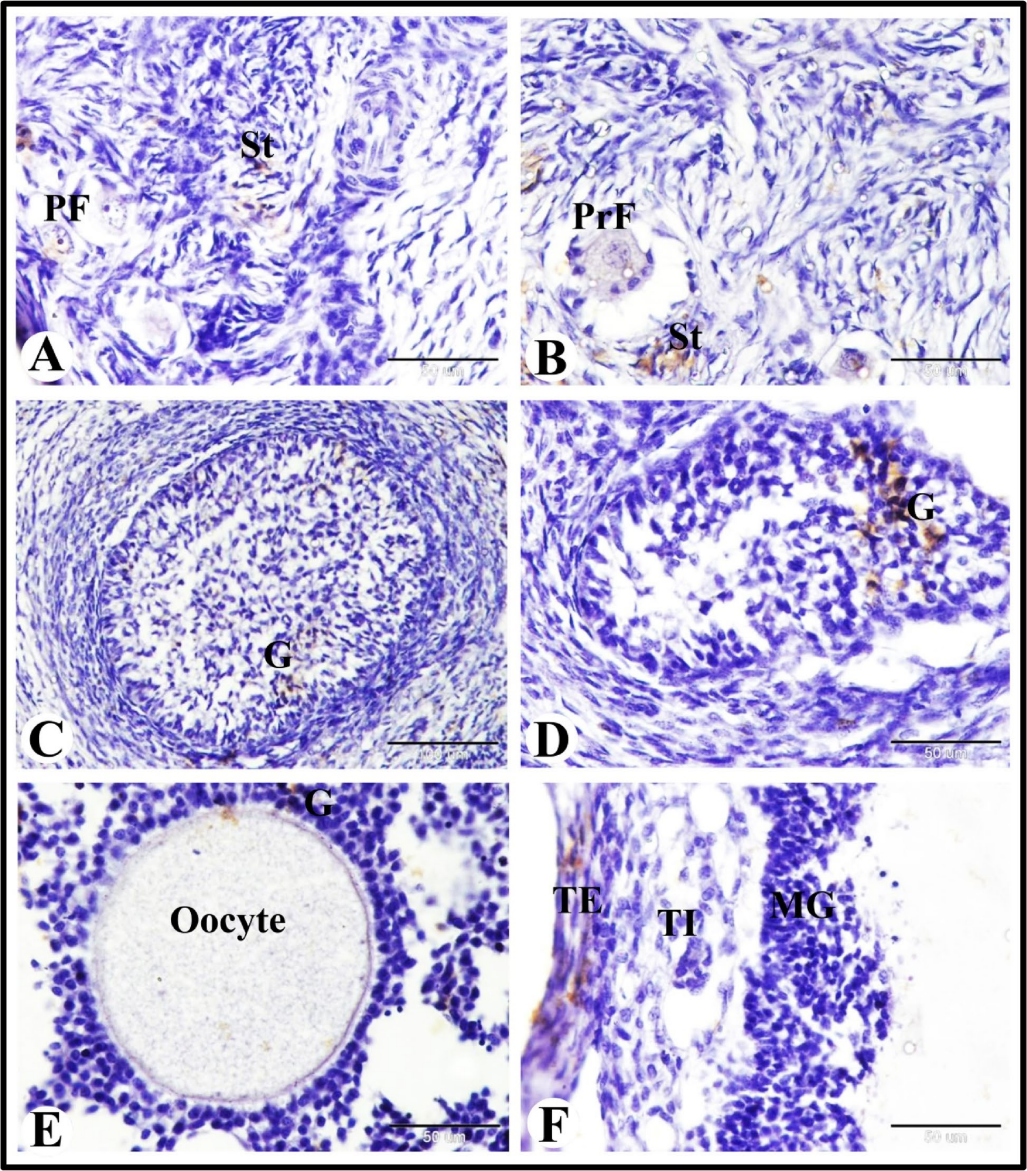
Photomicrograph of SOD2 immunostaining in the sheep ovary during the follicular phase. **A** & **B** Showing slight SOD2 immunostaining in the stroma cells (St) and negative SOD2 immunostaining in the primordial (PF) and primary (PrF) follicles. **C**-**E** Showing moderate SOD2 immunostaining in the granulosa cells (G) of the growing (**C** & **D**) and antral (**E**) follicles and negative SOD2 immunostaining in the oocyte. **F** Showing moderate SOD2 immunostaining in theca externa cells (TE) and negative SOD2 immunostaining in the granulosa cells (**G**) and theca interna cells (TI) of preovulatory follicles. Original magnification; **A**-**B** & **D**-**F**: X400, scale bar = 50 µm, C: X200, scale bar = 100 µm

We observed slight SOD2 immunostaining in the stroma cells and negative SOD2 immunostaining in the primordial and primary follicles (Fig. 7A and B). Moderate SOD2 immunostaining in the granulosa cells of the growing were also observed (Fig. 7C and D). While in the antral follicles we obseved moderate SOD2 immunostaining in the granulosa cells and negative SOD2 immunostaining in the oocyte (Fig. 7E). Whereas in the preovulatory follicles we found moderate SOD2 immunostaining in theca externa cells and negative SOD2 immunostaining in the granulosa cells and theca interna cells (Fig. 7F).

### PRA immunoexpression in the sheep ovary during the follicular phase of estrous cycle

**Fig. 8:**
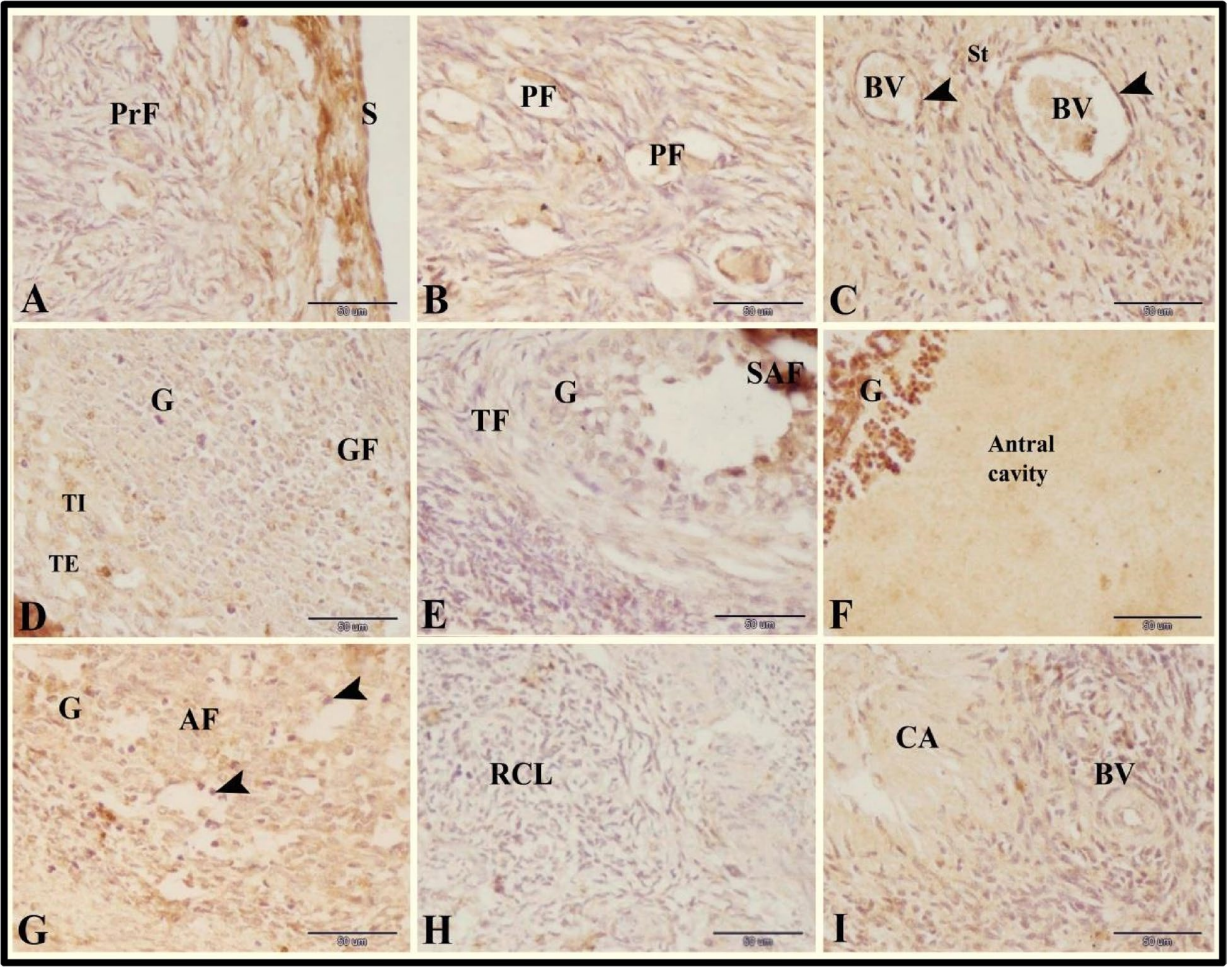
Photomicrograph of PRA immunoexpression in the sheep ovary during the follicular phase. **A** Showing strong PRA immunoexpression in the surface epithelial cells (S) and slight PRA immunoexpression in the primary (PrF) follicles. **B** slight PRA immunoexpression in the primordial (PF). **C** Showing strong PRA immunoexpression in the endothelial cells (arrowheads) of blood vessels (BV) and moderate PRA immunoexpression in the stroma cells (St). **D** Showing slight PRA immunoexpression in the granulosa cells (G), theca interna cells (TI) and theca externa cells (TE) of the growing follicles (GF). **E** Showing slight PRA immunoexpression in the granulosa cells (G) and theca folliculi cells (TF) of the small antral follicles (SAF). **F** Showing strong PRA immunoexpression in the granulosa cells (G) of the preovulatory follicles. Note the large antral cavity. **G** Showing slight PRA immunoexpression in the granulosa cells (G) and negative PRA immunoexpression in the apoptotic granulosa cells (arrowheads) of the atretic follicles (AF). **H** Showing negative PRA immunoexpression in the fibrous tissue (fibrocytes and collagen fibers) of the regressed corpus luteum (RCL). **I** Showing slight PRA immunoexpression in the fibrous tissue (fibrocytes and collagen fibers) and blood vessels (BV) of the corpus albicans (CA). Original magnification; **A**-**I**: X400, scale bar = 50 µm

Our results showed strong PRA immunoexpression in the ovarian surface epithelial cells, in the endothelial cells of blood vessels, and in the granulosa cells of the preovulatory follicles. We observed moderate PRA immunoexpression in the stroma cells. Also we found slight PRA immunoexpression in the oocytes and follicular cells of the the primordial and primary follicles, in the granulosa cells of the atretic follicles. In addition to slight PRA immunoexpression in the granulosa cells, theca interna cells and theca externa cells of the growing follicles and small antral follicles and in the fibrous tissue (fibrocytes and collagen fibers) and blood vessels (BV) of the corpus albicans. However, negative PRA immunoexpression in the apoptotic granulosa cells of the atretic follicles and in the fibrous tissue (fibrocytes and collagen fibers) of the regressed corpus luteum were demonstrated (Fig. 8A-I).

### Immunofluorescence of TUNEL assay in the sheep ovary during the follicular phase of estrous cycle

**Fig. 9:**
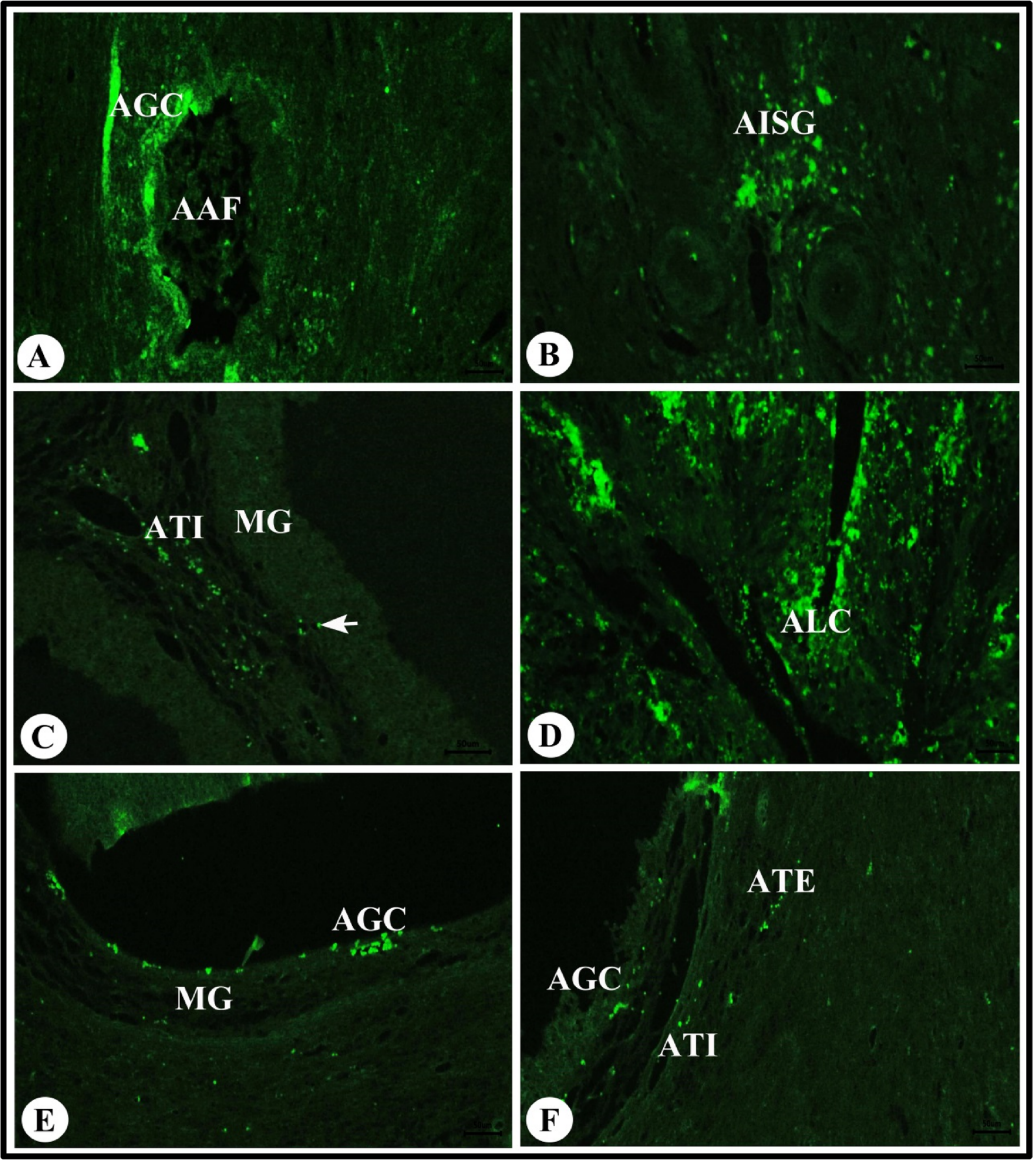
Photomicrograph of immunofluorescence of TUNEL assay in the sheep ovary during the follicular phase. **A** Showing apoptotic granulosa cells (AGC) in the atretic antral follicle (AAF). **B** Showing apoptotic interstitial gland cells (AISG). **C** Showing apoptotic basal granulosa cells (arrow) of membrana granulosa (MG) and apoptotic theca interna cells (ATI) of atretic grafiaan follicles. **D** Showing apoptotic lutein cells (ALC) of the regressed corpus luteum. **E** Showing apoptotic apical (antral) granulosa cells (AGC) of membrana granulosa (MG) of atretic grafiaan follicles. **F** Showing apoptotic granulosa cells (AGC), apoptotic theca interna cells (ATI) and apoptotic theca externa cells (ATE) of atretic grafiaan follicles. Original magnification; **A**-**F** X400, scale bar = 50 µm

Our results showed apoptotic granulosa cells in the atretic antral follicle (Fig. 9A) and apoptotic interstitial gland cells (Fig. 9B). We also observed apoptotic lutein cells in the regressed corpus luteum and apoptotic apical (antral) and basal granulosa cells of membrana granulosa, apoptotic theca interna cells and apoptotic theca externa cells of atretic grafiaan follicles (Fig. 9C-F).

**Fig. 10:**
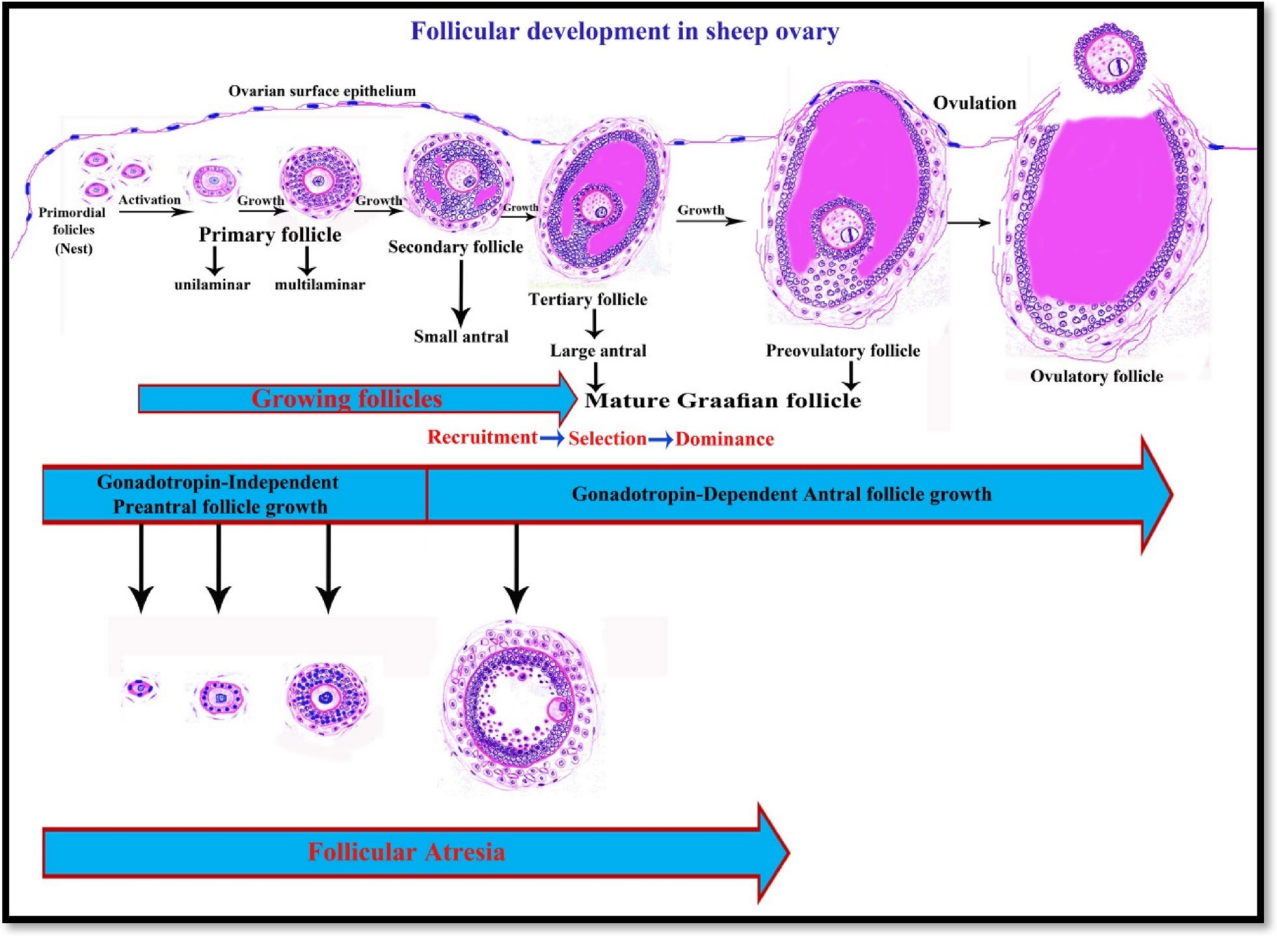
Drawing diagram showing the follicular development and follicular stages in sheep ovary. *Preantral phase*: Formation of primordial follicles which present in nests; Primordial follicle has a single layer of flattened follicular cells. Follicular activation of the primordial follicles to form unilaminar primary follicles; unilaminar primary follicle has a single layer of cuboidal follicular cells. Then growth of unilaminar primary follicles to form multilaminar primary follicles; multilaminar primary follicles have several layers of granulosa cells and few theca cells. All the preantral follicles have a primary oocyte surrounded by follicular cells and separated from the follicular cells by increased thickness zona pellucida. *Antral phase*: Follicle growth continues through the phases of recruitment, selection, dominance, and preovulatory stage of follicular waves. Secondary follicle has several layers of granulosa cells with small accumulations (pools) of follicular fluid in the intracellular spaces and a small number of theca cells. Tertiary follicle has several granulosa cell layers, corona radiata, cumulous oopherous, theca folliculi, and primary oocyte and is characterized by a single large antral cavity which contains follicular fluid. Preovulatory (Mature Graafian) follicle is the last stage of follicle development; these follicles are larger, have more antral fluid and may contain a secondary oocyte and protrude from ovarian surface epithelium and separated from them by very thin layer of granulosa cells and tunica albuginea. Ovulatory follicle is preovulatory from which the ovum and its surrounding cells released to the peritoneal catvity by rupture of the thin follicular wall

Herein we drawing a diagram to give a new schematic sequence of ovarian follicles development in Saidi sheep and describing the features of different follicular types (Fig. 10).

## Discussion

The estrous cycle is the interval in days between the start of two different periods of estrus, or heat. The estrous cycle is regulated by a complex system involving a number of hormones and factors. The hypothalamic gonadotropin-releasing hormone (GnRH) stimulates the pituitary gland to secret luteinizing hormone (LH) and follicle-stimulating hormone (FSH), prompting the ovaries to grow follicles [33]. Saidi sheep had nearly no reproductive seasonality and the estrous cycles were distributed along the year but a decline in the estrous and ovarian activity were observed during spring [5].

Our results revealed that the Saidi ewe ovary during the follicular phase of the estrous cycle contained many follicles in different stages of development and atresia in addition to regressed corpus luteum (corpus albicans). During folliculogenesis the flattened epithelium (pre-granulosa cells) which surrounded the oocyte in each primordial follicle differentiates to form the granulosa cells (GCs). The most of dormant primordial follicles remain quiescent and die. Those that survive serve as the ovarian follicle reserve with only a minority activated to transform into primary follicles. These follicles recruited to develop and establish the pool of growing follicles. The transition into primary follicles is characterized by a morphological change of the GCs, from flattened to cuboidal cells [34]. Oxidized glutathione (GSSG) is recycled by the enzyme glutathione reductase (GR). It converts it to a reduced form (GSH) in an NADPH-dependent manner. In the ovary, the strongest reactivity to the antibody was observed in oocytes, followed by granulosa cells, corpus luteum, and interstitial cells. Gamete viability and efficiency of fertility is increased by GSH. It is expressed in these tissues and is predicted to play a pivotal role in the reproduction process as a source of GSH [35].

We observed that the growing follicles were formed of primary occyte surrounded by more than one layer of follicular cells. The changes in the oocyte during follicular growth included: the occyte increased in size, Yolk granules appeared in the cytoplasm of the oocyte and Zona pellucida appeared around the oocyte. While the change in the follicular cells included: the flattened follicular cells change into cuboidal or columnar cells. The cuboidal cells were divided by mitotic divisions, increased in number and formed a stratified epithelium around the oocyte. The small antral follicles were growing follicles contained one or more small pools filled with follicular fluid. The mature Graafian follicles were follicle contained a large single antrum. Oocytes secrete GDF9 (growth differentiation factor 9) and BMP15 (bone morphogenetic protein 15) which mainly regulates its growth and maturation as well as granulosa proliferation and differentiation. AMH (anti-Mullerian hormone), inhibin, activins, and TGFα (transforming growth factor-alpha) are other paracrine factors which also acts as regulators of these processes, they are produced by the somatic cells surrounding oocytes [36]. A changing intrafollicular microenvironment that controls primordial follicle growth into a cohort of growing follicles is created by bidirectional somatic cell–oocyte signaling. From this one antral follicle is selected to ovulate a healthy oocyte. These intercellular communications allow the oocyte to determine its own fate by influencing the intrafollicular microenvironment. This provides the necessary cellular functions for oocyte developmental competence. This is defined as the ability of the oocyte to complete meiosis and undergo fertilization, embryogenesis, and term development. These coordinated somatic cell–oocyte interactions balance cellular metabolism that requires energy during folliculogenesis. This includes changing energy utilization during meiotic resumption [36].

Antral follicle recruitment and oocyte meiotic resumption is a two-way bidirectional paracrine communication between oocyte and cumulus cells [37]. Development of mammalian ovarian follicles is orchestrated and coordinated by oocytes. Normal follicular differentiation and the production of an oocyte competent to undergo fertilization and embryogenesis are regulated by an oocyte-granulosa cell regulatory bidirectional loop. This interplay is essential for oocyte development as well as for follicular development [9, 38, 39]. In sheep, emergence and growth of ovarian antral follicles in follicular waves does not require changes LH secretion and involves changes in the follicular sensitivity to LH and the largest follicle of a wave has limited effects on other small follicles and on the time of emergence of the next follicular wave. Hence functional dominance which is seen in cattle, is absent in sheep. For periodic peaks with endogenous rhythm in serum FSH concentrations is independent of ovarian follicular dynamics exists in sheep [40, 41]. The expression patterns of most steroidogenic enzymes, in the theca and granulosa compartments of antral follicles increase in each follicular wave in the ewe, paralleled with serum estradiol concentrations. The largest follicle of any follicular wave can be ovulate if a gonadotropin surge is provided [42].

Our results revealed that the preovulatory follicles were large antral follicles with a very large single antrum and thin wall and bulging from the ovarian surface. The wall of preovulatory follicles showed dissociated granulosa cells, thin follicular basement membrane, theca interna and theca externa. Ovulatory follicle selection in sheep is a passive process. Here the largest follicle of the cohort of recruited follicles inhibits the FSH support to the other follicles. This is done via negative feedback action of its estradiol and inhibin. Maintenance of the dominant follicle while the others regress could be related to an autocrine stimulation of the dominant follicle by insulin like growth factor 1 (IGF1) together with the differentiation of LH receptors [13]. The granulosa cells and oocyte secret follicular fluid which is a plasma exudate [34].

Our findings revealed that ovarian tissue expressions of GR and SOD2 in addition to increased level of serum antioxidant prameters were involved in follicular development and follicular and corpus luteum regression. Reactive oxidative species (ROS) are created in ovarian follicles during steroidogenesis and induce lipid peroxidation [43, 44]. Antioxidants have to constantly minimize the harmful effects of ROS to preserve cell function [44]. The Superoxide dismutase (SOD) family is a crucial enzyme in antioxidant defense since it promotes the breakdown of superoxide into hydrogen peroxide and oxygen molecules and serves an important role in maintaining cellular ROS balance. Glutathione peroxidases (GPx) convert hydrogen peroxide to H2O and O2 while simultaneously oxidizing glutathione. On the other hand glutathione reductase (GR) converts the oxidized glutathione (GSSG) to its reduced form (GSH) in an NADPH-dependent way [44]. GSH/GSSG is the most significant redox pair, and it is crucial for antioxidant defense and the regulation of pathways important for overall body homeostasis. An increase in the redox ratio, as reflected by GSH/GSSG, indicates decreased oxidative stress [45, 46]. Only by maintaining an appropriate balance of those enzymes can optimal protection be achieved. Superoxide dismutase exists in mammals in three different isoforms: SOD1 (cytoplasmic), SOD2 (mitochondrial), and SOD3 (extracellular). However, the only isoform required for the continual existence of aerobic organisms is SOD2 [47, 48]. Meanwhile, SOD2 plays a crucial role in controlling a wide range of physiological functions, including immunological response, and protects mitochondria against ROS. ROS and antioxidants are essential for female reproductive processes. In processes associated with reproduction such as follicular development, oocyte maturation, luteolysis, and progesterone production by the corpus luetum the balance between ROS and antioxidants is essential [49].

It was found that the concentrations of many of enzymatic antioxidants vary according to the follicle size and the stage of the estrous cycle in buffaloes. This suggesting their possible role in the process of follicular development during estrous cycle [50]. There was a close relation between the increased ovarian activity and antioxidant indices during estrous cycle in sheep [51].

Ovulation is a self-controlled inflammatory process triggered by a pulsatile release of gonadotropin-releasing hormone (GnRH) from the hypothalamus to initiate the ovulatory surge of luteinizing hormone and follicle-stimulating hormone [49]. GnRH induces the expression of two key genes, progesterone receptor (*Pgr*) and prostaglandin-endoperoxide synthase 2 (*Ptgs2*), in the granulosa cells of preovulatory follicles. Their gene products PGR and PTGS2 activate two separate pathways that are both essential for successful ovulation. PGR plays an essential role in ovulation: it terminates ovulatory inflammation by diminishing the gonadotropin surge-induced *Ptgs2* expression [52]. Ovulation involves the activity of SOD. LH stimulates SOD to produce hydrogen peroxide, which acts as a substrate for peroxidase to promote progesterone synthesis via a free radical process. This causes a cyclic variation in the amount of SOD during the reproductive cycle, with a high level during proestrus [49]. In the present study, immunostaining for SOD2 was negatively intense in primordial follicles, primary follicles, and oocytes of follicular phase ovaries while moderately intense in growing, antral and preovulatory follicles. Besides that, in a rabbit ovary in vitro investigation, superoxide radical formation was found to be important in follicular rupture and was suppressed by SOD, which was thought to be involved in progesterone production [49]. Female mice lacking SOD are either subfertile or infertile, with ovarian abnormalities and reduced FSH and LH hormone levels [49]. As a result, it may be deduced that, SOD is essential to maintain normal ovulatory function. SOD2 mRNA was expressed during proestrus and then decreased as the estrous cycle progressed. SOD2 expression in normal mice increased significantly during proestrus and thereafter dropped [49].

The improved GSSG to GSH transition, which results in a higher concentration of free GSH, is predicted by the increased GR activity. GR activity is crucial for cellular defense against oxidative stress and toxic chemicals. GSH is known to increase gamete viability and fertility, hence GR indirectly aids in the protection of cells from oxidative stress and cytotoxic agents. GR appears to play a crucial role in the female reproductive system. The resultant GSH appears to have many functions, including protecting embryos from oxidative stress and maintaining oocyte potency through antioxidative and redox enzymes [35].

Less intense GR immunostaining was seen in the main follicular oocyte of follicular phase ovaries, moderately intense staining was shown in apoptotic granulosa cells, and the strongest staining was seen in the lutein cells of the regressed corpus luteum. These results are in line with those of other studies in which immunostaining for GR in the ovary was present in preantral and antral follicles with intense staining in corpus luteum and granulosa cells [35]. Since excessive SOD2 can enhance superoxide generation, which is linked to proliferation and metastasis in aggressive tumors [53, 54], it has been previously noted that excessive SOD2 leads to ovarian malignancies [55]. The activation of numerous signaling pathways, such as protein kinase B, nuclear factor-kB, calcineurin, c-Jun N-terminal kinase, and mitogen-activated protein kinase, however, occurs when the GSH/GSSG redox is shifted toward the oxidizing state [45, 46].

TUNEL assay was performed in the sheep ovary during the follicular phase to evaluate if SOD2 activity is involved in proliferation and normal apoptosis. Positive apoptotic expression was observed in atretic grafiaan follicles (interstitial gland cells, granulosa cells, theca interna cells, theca externa cells) as well as granulosa cells in regressed corpus luteum lutein cells. As a result, SOD2 activity was found to be within the normal physiological range.

In the current study, the levels of glucose and urea in saidi ewe were lower than their concentration in Ossimi sheep however the concentrations of cholesterol, total protein, AST, and ALT were higher this difference may be contributed to different breeds [56].

In both buffalo and mares, TAC and MDA showed low concentrations during the follicular period [57, 58]. While NO showed a high level. Due to its multifarious involvement in vasodilation, regulation of follicular basement membrane permeability, steroidogenesis, and ovulation, as well as NO, a key intra-ovarian factor, controls the process of follicular development [59]. Similar results were obtained in the current study.

There has been no significant variation in serum levels of albumin, globulin, and total protein during the estrous cycle. Glucose, cholesterol, and triglyceride levels, on the other hand, tended to be significantly higher during the estrous phase [57]. Because glucose is the ovary's primary energy source, it plays a crucial role in ovarian metabolism. The serum glucose level was considerably greater in the estrous phase. A similar finding was found in the mare [57]. Maintaining physiological glucose levels is required for mare fertility and reproductive success [60]. Here, the serum levels of cholesterol dramatically rise during estrous phase as steroid hormones (progesterone and estrogen) are synthesized from cholesterol [61].

The role of PRA has been explored as a regulator of uterine function as previously reviewed [62, 63]. PRA receptor has been identified in the gonads of several species including the ovaries of rabbits [17, 64], porcine [65], cows [66] and mice [67]. The present study aims to compare immunostaining for PRA between follicle sizes during the follicular phase. In the present study, the intensity of staining for PRA increased as follicle size increased. This may indicate a role for PRA in late-stage follicular growth, maturation, and/or ovulation. The findings revealed that PRA immunostaining was most intense in preovulatory antral follicles during the follicular phase.

Immunostaining of PRA in the ewe ovary during the follicular phase revealed most intense in the surface epithelial cells, granulosa cells of the preovulatory follicles, endothelial cells of blood vessels, moderate intense in the stroma cells as well as less intense in the primordial, primary follicles, granulosa cells, theca interna cells and theca externa cells of the growing follicles, the granulosa cells and theca follicle cells of the small antral follicles, in the fibrous tissue (fibrocytes and collagen fibers) and blood vessels of the corpus albicans. Moreover, negative PRA immunoexpression in the apoptotic granulosa cells of the atretic follicles and fibrous tissue (fibrocytes and collagen fibers) of the regressed corpus luteum. These findings are consistent with other findings in buffalo ovarian follicles [68] in which immunostaining for primary follicles, was present in preantral and antral follicles with intense staining in granulosa cells and primary follicles. In the bovine ovary, the immunoreactive staining pattern is consistent with the findings of the current study [66]. Moreover, The expression of PGR mRNA in follicles and theca interna tissue increases continuously with the development of the follicles in the bovine ovary during the estrous cycle [69].

The mean level of plasma estradiol during the follicular phase was 2.32 ± 0.08 pg/ml, while the average level of plasma progesterone was 0.86 ± 0.06 ng/ml. The recorded hormonal levels were lower than those described in Gaddi Sheep [70]. During the follicular growth, the concentration of E2 increases progressively as the follicle grows, although P4 was decrease. A similar finding was achieved in mare [57]. The results of the receptor and hormone study demonstrated that there was more PR expression during estradiol dominance. The current results support the theory that estrogen stimulated the expression of these receptors [68]. There is a negative association between plasma progesterone levels and the PR score for all ovarian cell types in bovine ovaries, suggesting that estradiol upregulates the expression of these receptors while progesterone hormone downregulates them [66].

Our study revealed that the follicular atresia in the sheep ovary during the follicular phase of estrous cycle were most prominent in the antral follicles especially the large antral ones. Atretic large antral follicle was characterized by the presence of apoptotic oocyte, apoptotic basal and antral granulosa cells, thickening and proliferation of theca interna, and recruitment of several macrophages which were phagocytosing dying granulosa cells. These results were similar those observed in bovine [71] and rabbit [17]. The intrafollicular macrophages were considered to play an important role in the dynamics of the follicle, especially during both its development and atresia as it phagocytosed apoptotic granulosa cells [72]. Follicular atresia is a cell death event that occurs in the great majority of follicles before ovulation in the mature mammalian ovary. It is mainly occurs via apoptosis and there are a possible cooperating role of autophagy and apoptosis in follicular atresia and/or follicular survival. Autophagy is a cellular and energy homeostatic response that maintain the number of healthy follicles, germ cell survival, and removal of corpus luteum remnants [73, 74].

Herein we found that at early stage of the antral follicles atresia the PAS positive follicular basement membrane and zona pellucida became thickened. While in the terminal stage basement membrane became hyalinized and formed collapsed PAS positive glassy membrane. It is concluded that the basal lamina was not degraded upon atresia, but does undergo a variety of other changes as loopy, folding and collapse [75]. In some cases of the large antral follicles atresia the basement membrane became thin and interrupted and zona granulosa became formed of single layer. The basal lamina was often penetrated by macrophages and invading capillaries [71].

## Conclusion

The most prominent features of the follicular phase of estrous cycle in Saidi Egyptian sheep were the presence of Graafian and preovulatory follicles and regressed corpus luteum. We also concluded that this ovarian histological picture was controlled by hormonal, oxidative and blood metabolites factors. The findings of our study are increasingly important to understanding the reproduction in Saidi sheep which assist in improving the reproductive outcome in this breed. Moreover, our results may help for implementation of a genetic improvement program and utilizing the advanced reproductive techniques as estrous synchronization, artificial insemination and embryo transfer.

### Data availability

All data generated or analysed during this study are included in this published article.

## Data Availability

The datasets used and/or analysed during the current study are available from the corresponding author on reasonable request.
